# Supplementary material for: Entamoeba histolytica “mutator” strain with a high rate of genetic mutations assists the elucidation of drug resistance mechanisms
Source: Microbiol Spectr. 2025 Jun 12;13(8):e01210-25. doi: 10.1128/spectrum.01210-25 (PMC12323330; doi:10.1128/spectrum.01210-25)
Supplement: Supplemental material — Supplemental methods; Fig. S1 to S10. [file spectrum.01210-25-s0001.pdf]

Supplementary Material

MATERIALS AND METHODS

Quantitative real-time PCR

Total RNAs were isolated from 5 × 10^5 cells using RNeasy Plus Mini Kit (QIAGEN, Hilden, German). cDNAs were obtained using oligo-dT using Super Script IV Reverse Transcriptase (Thermo Fisher Scientific, USA) according to the manufacturers protocol. mRNA expression level of mock and EhMutator strains in the presence or absence of tetracycline was evaluated by quantitative RT-PCR as previously described (1, 2) with some modifications. RNA polymerase II gene was used as an internal control (GenBank accession number, XP_649091). The primer sequences were shown in Table S6. The PCR reaction was performed using the QuantStudio 7pro PCR system (Thermo Fisher Scientific, USA). The thermal condition of the reaction was as follow 95°C for 20 s, followed by 40 cycles of 95°C for 1 s and 60°C for 20 s. A final step at 95°C for 1 s, 60°C for 20 s, and 95°C for 1 s was used to remove primer dimers.

Subcellular fractionation of the transformants

The subcellular fractionation was conducted according to a previously reported method, with some modifications (1). Briefly, ~3 × 10^5 trophozoites were washed with cold PBS containing 2% glucose, resuspended in a homogenization buffer (250 mM sucrose, 50 mM Tris [pH 7.5], 50 mM NaCl, and 0.1 mg/mL E-64), and homogenized on ice with a Dounce homogenizer. Afterwards, the unbroken cells were removed by centrifugation at 400 × *g* for 2 min. Then, the supernatant was centrifuged at 13,000 × *g* at 4°C for 10 min to obtain the pellet (p13) and the supernatant (s13) fractions. The s13 fraction was further separated by centrifugation at 100,000 × *g* at 4°C for 1 h to yield the soluble (s100) and the pellet (p100) fractions. These fractions were analyzed by immunoblot with anti-FLAG M2 (Sigma-Aldrich, No F1804), anti-CPBF1 (3), or anti-CS1 (4), and then with anti-rabbit or anti-mouse IgG-conjugated HRP antibodies (Cell Signaling Technology, Inc. Danvers, MA, USA).

Indirect immunofluorescence assay

An indirect immunofluorescence assay was conducted as previously described (5). Trophozoites were attached to 8-mm-round wells on a glass slide, fixed with 3.7%

paraformaldehyde, permeabilized with 0.1% Triton X-100 for 10 min, and treated with anti-FLAG antibodies. Alexa 488-conjugated IgG (Molecular Probes, Eugene, OR, USA) was used as the secondary antibody. Nuclei were stained with 4′,6-diamidino-2-phenylindole (DAPI). Images were acquired using an LSM780 confocal laser-scanning microscope (Carl Zeiss AG, Oberkochen, Baden-Württemberg, Germany).

Doubling time

Transformant trophozoites introduced with pMock or pNLS-FLAG-EhPolDel^{AA} were inoculated at 1,600 cell/ml in 6 ml of BI-S-33 medium containing 6 μg/mL G418 in the presence or absence of 10 μg/mL tetracycline. The parasite number was counted every 24h hours.

TABLES

TABLE S1 Summary of the HiSeqX sequencing used in this study

TABLE S2 List of SNPs identified from the mock strains

TABLE S3 List of SNPs identified from the mutator strains

TABLE S4 Number of SNP per contig size

TABLE S5 Candidate genes responsible for the miltefosine-resistance

TABLE S6 List of oligonucleotides used in this study

REFERENCES

1. Saito-Nakano Y, Mitra BN, Nakada-Tsukui K, Sato D, Nozaki T. 2007. Two Rab7 isotypes, EhRab7A and EhRab7B, play distinct roles in biogenesis of lysosomes and phagosomes in the enteric protozoan parasite *Entamoeba histolytica*. Cell Microbiol 9:1796-808.
2. Gilchrist CA, Houpt E, Trapaidze N, Fei Z, Crasta O, Asgharpour A, Evans C,

Martino-Catt S, Baba DJ, Stroup S, Hamano S, Ehrenkaufer G, Okada M, Singh U, Nozaki T, Mann BJ, Petri WA, Jr. 2006. Impact of intestinal colonization and invasion on the *Entamoeba histolytica* transcriptome. Mol Biochem Parasitol 147:163-76.

3. Furukawa A, Nakada-Tsukui K, Nozaki T. 2012. Novel transmembrane receptor involved in phagosome transport of lysozymes and beta-hexosaminidase in the enteric protozoan *Entamoeba histolytica*. PLoS Pathog 8:e1002539.
4. Okada M, Huston CD, Oue M, Mann BJ, Petri WA, Jr., Kita K, Nozaki T. 2006. Kinetics and strain variation of phagosome proteins of *Entamoeba histolytica* by proteomic analysis. Mol Biochem Parasitol 145:171-83.
5. Saito-Nakano Y, Yasuda T, Nakada-Tsukui K, Leippe M, Nozaki T. 2004. Rab5-associated vacuoles play a unique role in phagocytosis of the enteric protozoan parasite *Entamoeba histolytica*. J Biol Chem 279:49497-507.

Fig S1, Saito-Nakano et al.

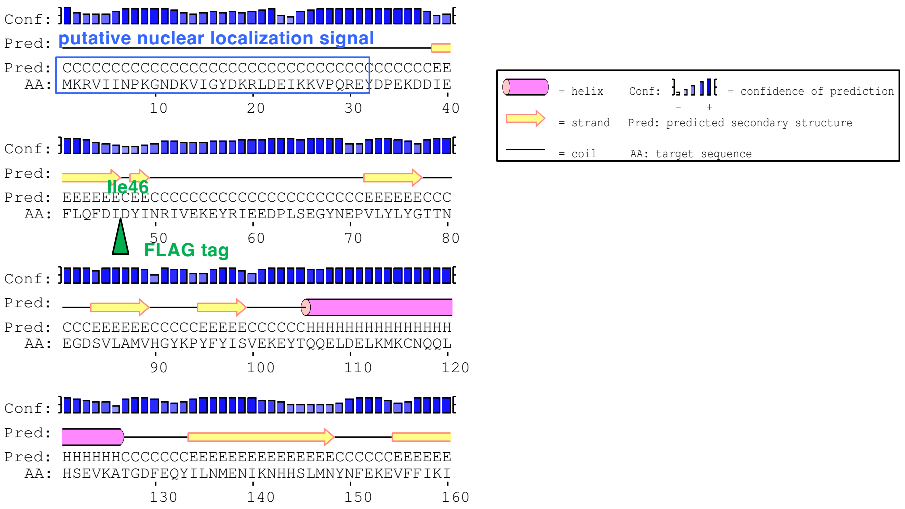

Fig S1 The position of FLAG-tagging in EhPolDel. The secondary structure of the N-terminal 160 amino acids of EhPolDel was predicted by the PSIPRED program (http://bioinf.cs.ucl.ac.uk/psipred/). The putative nuclear localization signal (NLS) predicted by cNLS Mapper (http://nls-mapper.iab.keio.ac.jp/cgi-bin/NLS_Mapper_form.cgi) with default parameters is shown by a blue rectangle. FLAG-tag was inserted after isoleucine at 46 amino acids (Ile46) and is depicted by a green arrowhead.

Fig S2, Saito-Nakano et al.

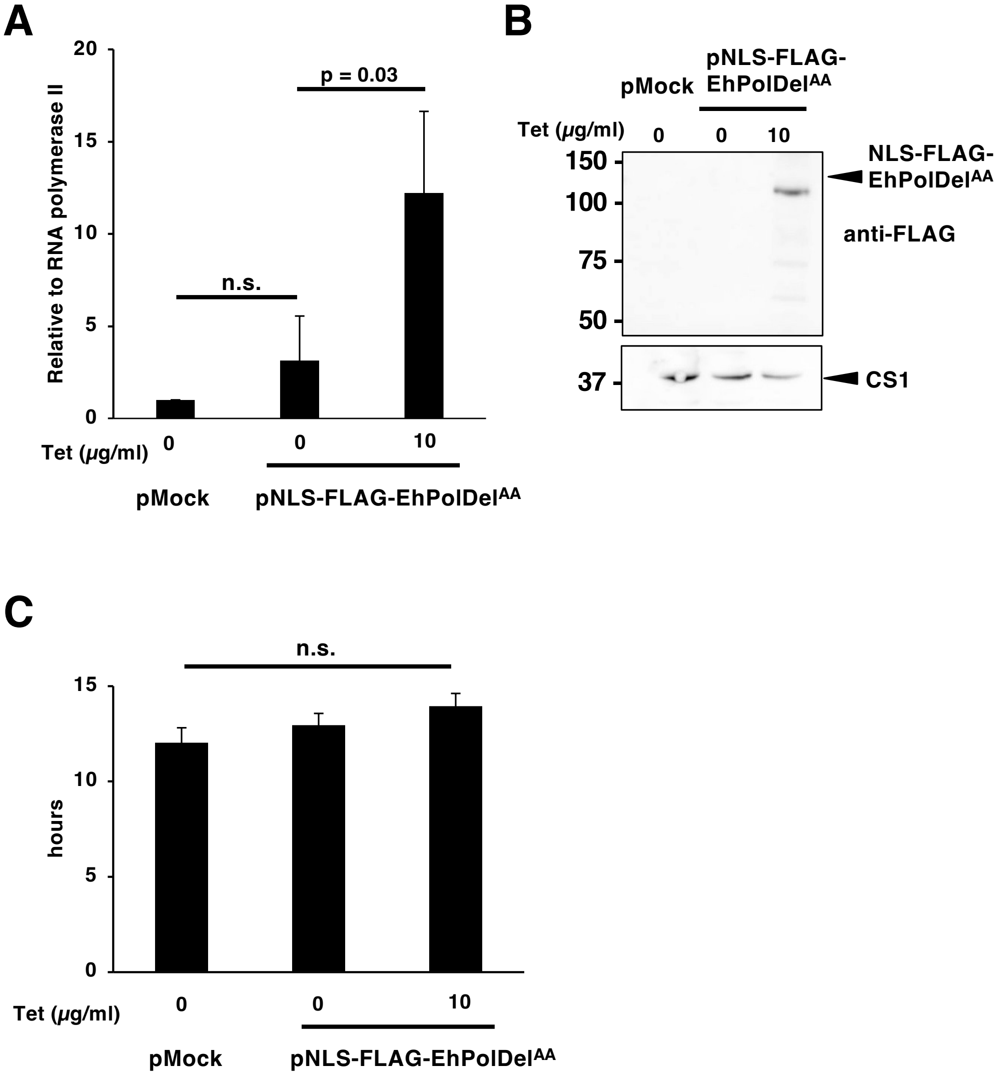

Fig S2 Tetracycline-dependent expression of NLS-FLAG-EhPolDel^{AA}. (A) The quantitative RT-PCR was performed using RNA from the amoeba transformants carrying either the pMock or pNLS-FLAG-EhPolDel^{AA} plasmid in the absence (Tet, 0) or presence (Tet, 10 μg/ml) of tetracycline for 18 hrs. The expression level of EhPolDel mRNA was normalized to that of RNA polymerase II, and relative values are presented. Error bars indicate the standard deviation from two independent experiments. (B) Expression of full length of NLS-FLAG-EhPolDel^{AA}. Transformant cells carrying the pNLS-FLAG-EhPolDel^{AA} plasmid were cultured in the presence of tetracycline for 18 hrs and subjected to immunoblot analysis using an anti-FLAG antibody. An anti-cysteine synthase (CS) antibody was used as internal control. Note that no band corresponding to NLS-FLAG-EhPolDel^{AA} was detected in the absence of tetracycline. (C) Expression of NLS-FLAG-EhPolDel^{AA} did not affect the amebic cell growth. Transformants were inoculated into 6 mL culture at 1600 cells/mL, and cell numbers were counted after 24 and 48 hrs. Data are means ± standard deviations of three independent replications. n.s., not significant by Student's t-test.

Fig S3, Saito-Nakano et al.

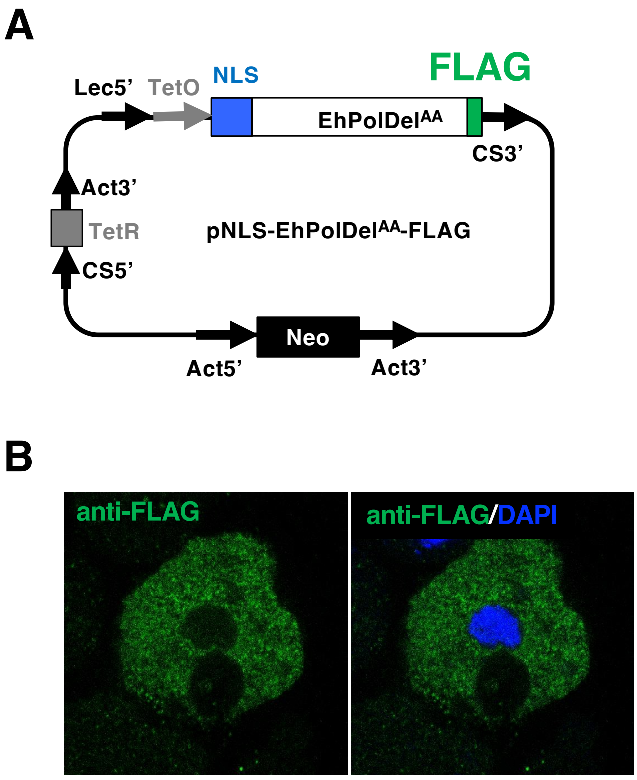

Fig S3 The importance of C-terminal domain of EhPolDel for the localization to the nucleus. (A) Schematic diagram of the plasmid construction for the expression of carboxy terminal FLAG-tagged EhPolDel^{AA}. (B) Failure of nuclear localization of EhPolDel^{AA} fused to FLAG tag at the C-terminus. Carboxy terminal FLAG-tagged protein was expressed in the amebas using the plasmid shown in (A) and subjected to the indirect immunofluorescence assay using an anti-FLAG-tag antibody. The FLAG signal (green) was excluded from the nucleus stained with DAPI (blue). The large dark region represents a vacuole.

Fig S4, Saito-Nakano et al.

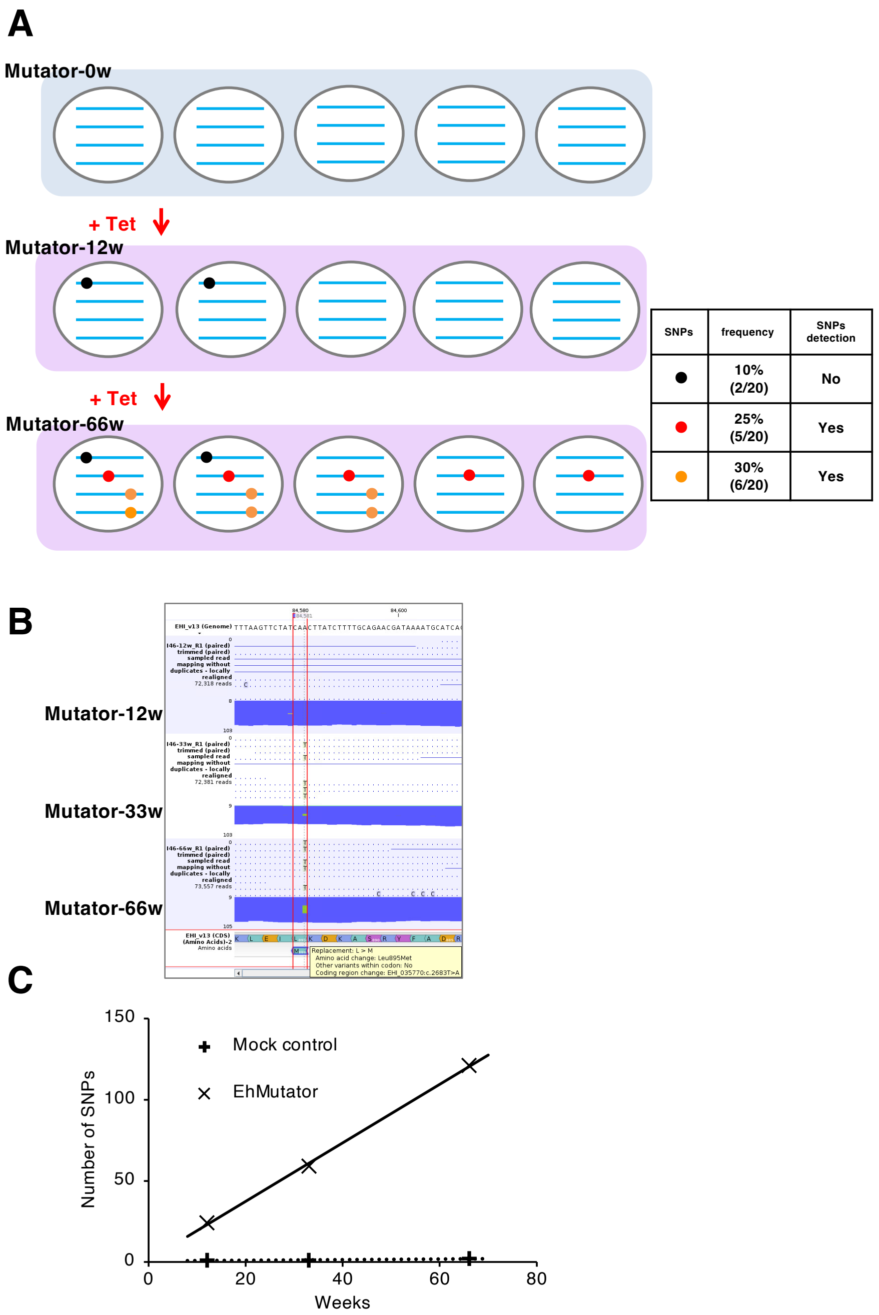

Fig S4 Detection and definition of SNPs in *E. histolytica* with a polyploid genome. (A) A schematic representation of detectable SNPs. Circles and blue lines in the circles represent trophozoites and four chromosomes in the trophozoites. SNPs present in a subset trophozoites, accounting for 10% of the read count (black dots), are not detectable (a middle panel). Detectable SNPs are those with an allele frequency more than 20% (red and orange dots). (B) A representable example of detection of EHI_035570^{T2683A}, where mutations were detected in Mutator-33w and -66w but not observed in Mutator-12w (see also Table S3). (C) Time-dependent accumulation of SNPs in EhMutator strains but not in mock strains.

Fig S5, Saito-Nakano et al.

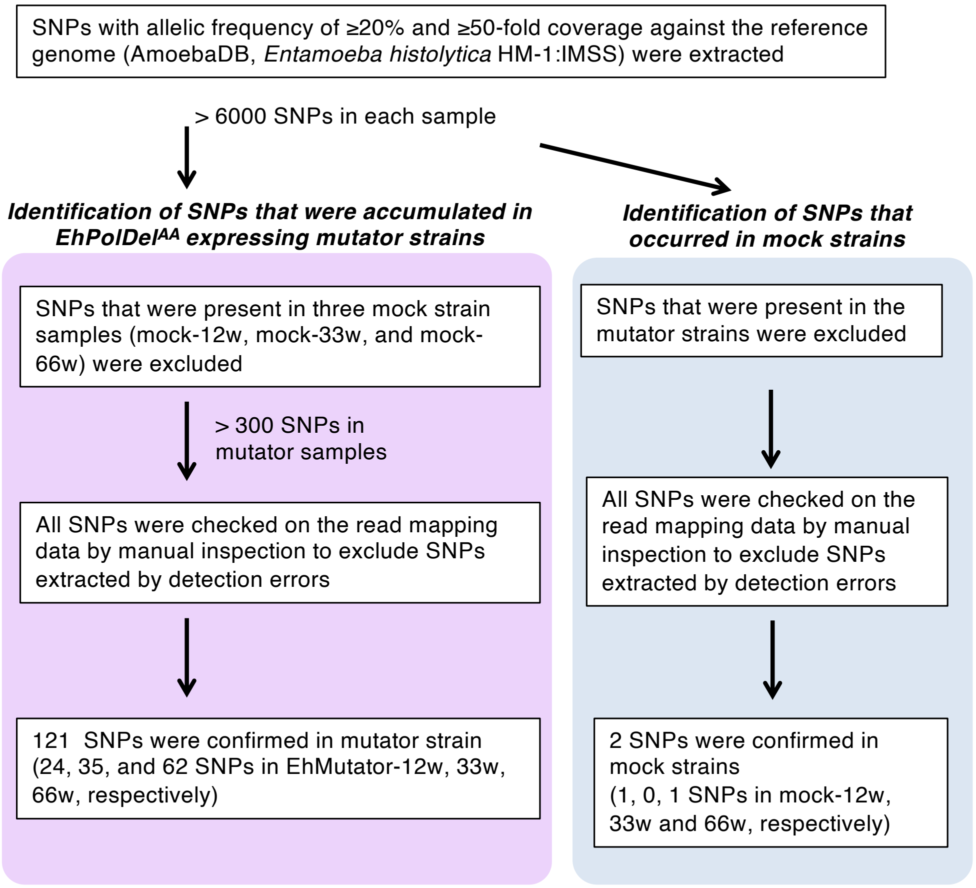

Fig S5 Flow diagram of SNP identification from HiSeq X sequencing data. A whole genome of EhPolDel^{AA}-expressing and mock control strains were sequenced by HiSeq X. All samples of EhMutator strain, harvested after tetracycline induction for 12, 33, and 66 weeks showed more than 6,000 SNPs. SNPs were defined as substitutions showing higher than 20% allelic frequency and higher than 50-fold coverage.

Fig S6, Saito-Nakano et al.

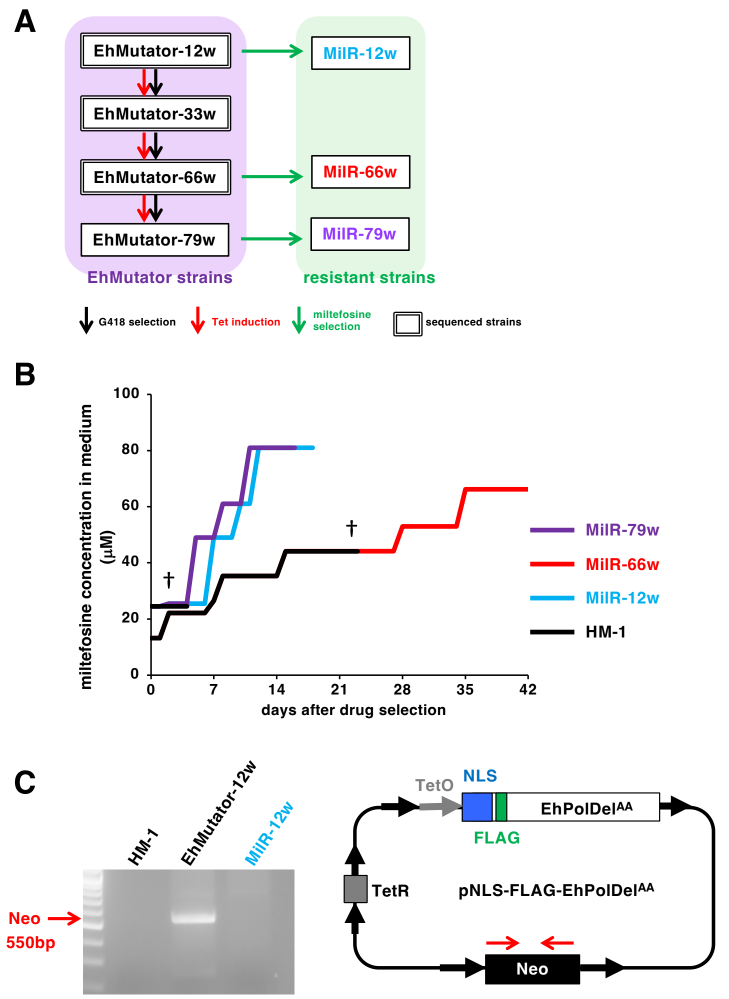

Fig S6 Profiles of generation of miltefosine-resistant strains from EhMutator-12w, -66w, and -79w. (A) A schematic diagram of generation of miltefosine-resistant strains. Three miltefosine-resistant strains, MilR-12w, MilR-66w, MilR-79w (a green rectangle), were generated from EhMutator-12w, EhMutator-66w, and EhMutator-79w (a purple rectangle), respectively. Miltefosine selection (green arrows) was carried out in the absence of G418 (black arrows) and tetracycline (red arrows) to prevent the emergence of additional SNPs. (B) Miltefosine concentrations in the course of resistance generation. Miltefosine selection was initiated at 13 μM for EhMutator-12w (a blue line) and parental HM-1 strain (black line), with the concentration gradually increased. By day 23, HM-1 strain had died, whereas MilR-12w continued to grow until day 42, at which point a resistant strain was isolated. MilR-12w was able to be maintained at 24 μM miltefosine. Miltefosine-resistant strains were also obtained from EhMutator-66w (a red line) and EhMutator-79w (a purple line) by gradually increasing the miltefosine concentration, starting at 24 μM. By day 14, the resistant strains MilR-66w and MilR-79w were isolated, while the parental strain had died by day 5. Both MilR-66w and MilR-79w were capable of growing at 120 μM miltefosine. "†" depicts death of parental HM-1 strain. (C) Miltefosine-resistant MilR-12w strain lost the region containing neomycin resistance gene. The DNA fragment corresponding to the *neo* gene was not amplified (red arrows) from either MilR-12w or HM-1 by PCR, while it was amplified from EhMutator-12w.

Fig S7, Saito-Nakano et al.

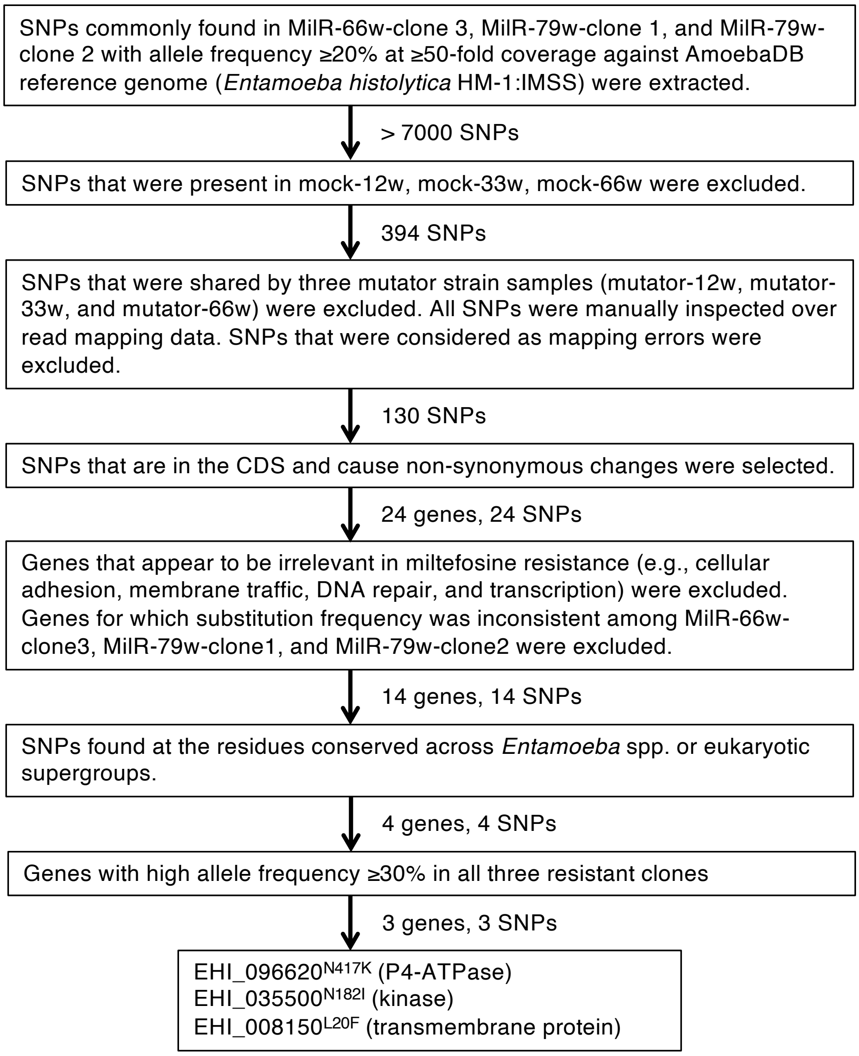

Fig S7 A flowchart of gene and SNPs identification associated with miltefosine resistance. SNPs commonly present in three miltefosine-resistant clones were extracted, and those found in mock and EhMutator strains were subtracted. After manually confirming the read mappings, 24 non-synonymous SNPs were identified in coding regions (see Table S4). Among these, four amino acids for which non-synonymous mutations were observed are conserved across the three *Entamoeba* species with available genome data (*E. moshkovskii*, *E. invadens, E. nuttalli*) as well as *Leishmania donovani*. Finally, SNPs with a low frequency (< 30%) were excluded. The SNPs causing EHI_096620^{N417K}, EHI_035500^{N182I}, EHI_008150^{L20F} were selected as candidate mutations associated with miltefosine resistance.

Fig S8, Saito-Nakano et al.

A

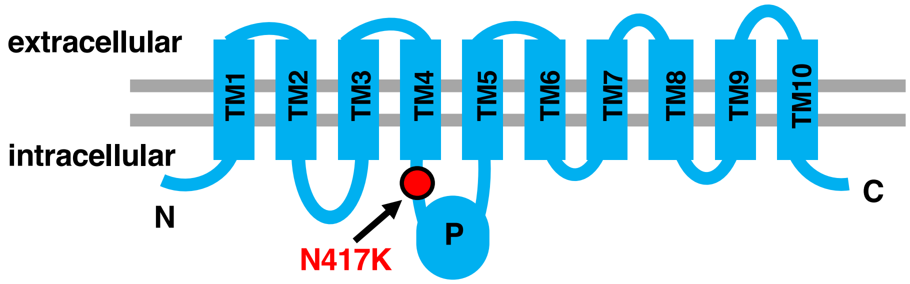

B

```
                                                                TM4
EHI_096620                      SLFLVRT--GRDSWYLDMEDISVG--KNAVQTFFRYFGLLSYLIPISCAVSLEVAKFIQT    364
ScDnf3_NP_013885                VLHKKKYIDQNKAWYLFQADA------GVAPTIMSFIIMYNTVIPLSLYVTMEIIKVVQS    528
HsATPase1A_XP_011511920         AIWNRRH--SGKDWYLNL---NYGGASNFGLNFLTFIILFNNLIPISLLVTLEVVKFTQA    371
LdATPase1_XP_003859368          VWWNSKY--RE-TPYLRFFISFRKNVTLWGYRYLSYFILLSYCVPISLFITIEVCKVVQA    378
                                     :        **                 : :: : .  :*:*  :::*: *. *:

EHI_096620                      MLMECDTDFNIYNIDEDGQLVTEKMAAKTSILNDELALVEYVLSDKTGTLTENSMVFKMA    424
ScDnf3_NP_013885                KMMEWDIDMYHAETN--T-----PCESRTATILEELGQVSYIFSDKTGTLTDNKMIFRKF    581
HsATPase1A_XP_011511920         YFINWDLDM-HYEP-TDT-----AAMARTSNLNEELGQVKYIFSDKTGTLTCNVMQFKKC    424
LdATPase1_XP_003859368          QWMRVDCLMMEYMSNRWR-----HCQPNTSNLNEQLAMVRFIFSDKTGTLTENVMKFKLG    433
                                  :. *  :                  .*: : ::*. * :::********   * *:

EHI_096620                      SVDGEVIEGKKLEENFKLYWNIDK-----------EKNG---------------------    452
ScDnf3_NP_013885                SLCGSSWLHNVDLGNSEDNFEDNRDNTNSLRLPPKAHNGSSIDVVSIGDQNVLDRLGFSD    641
HsATPase1A_XP_011511920         TIAGVAYGQNSQFGDEKTF-----------------------------------------    443
LdATPase1_XP_003859368          DALGNPIDADNLDECI--------------------------------------------    449
                                   *     .
```

Fig S8 The site of the identified mutation of EHI_096620 (P4-ATPase) and the protein alignment of homologs from *E. histolytica,* human, yeast, and *Leishmania donovani.* (A) Schematic diagram of the mutation site of EHI_096620 identified in this study. The mutated residue, N417K, is present in the intracellular region, and between the fourth transmembrane region (TM4) and ATPase catalytic domain (P). (B) Amino acid alignment of EHI_096620, yeast Dnf3, human ATPase1A, and *L. donovani* ATPase. The transmembrane region TM4 is indicated by a black horizontal line. The conserved asparagine residue is marked with a red arrow.

Fig S9, Saito-Nakano et al.

A

```
EIN_093630       IDSFQHRGLNGIHMCIVTQVGGSNLLSLIRLYHYRGIPLDITKEISKQVLIALNYLHTVC   174
EHI_035500       IDSFQHRGLNGIHMCIVTEVGGSNLLSLIKLYHYHGIPLEITKEISRQVLVALNYLHTKC   171
ENU1_094620      IDSFQHRGLNGIHMCIVTEVGGSNLLSLIKLYHYRGIPLEITKEISRQVLVALNYLHTKC   171
ScSky1_1Q8Y_A    LDHFNHKGPNGVHVVMVFEVLGENLLALIKKYEHRGIPLIYVKQISKQLLLGLDYMHRRC   151
HsSprk2_2X7G_A   IDDFKISGMNGIHVCMVFEVLGHHLLKWIIKSNYQGLPVRCVKSIIRQVLQGLDYLHSKC   160
                 :* *:  * **:*: :* :* * :**  *   .::*:*:  .*.* :*:* .*:*:*  *

EIN_093630       GLIHTDLKPENVLLNFIIDINHV------------------------------KKRSMVP   204
EHI_035500       SLIHTDLKPENVLLNFVIDHNHI------------------------------KRRNQVP   201
ENU1_094620      SLIHTDLKPENVLLNFVIDHNHI------------------------------KRRNQVP   201
ScSky1_1Q8Y_A    GIIHTDIKPENVLMEIVDS-----------------------------------------   170
HsSprk2_2X7G_A   KIIHTDIKPENILMCVDDAYVRRMAAEATEWQKAGAPPPSGSAVSTAPAADLLVNPLDPR   220
                  :****:****:*: .

EIN_093630       PAQNIQVMLADFGNANWVNERFTNDIQTRQYRCPEVMLGLHWGCPADIWSHACVIFELLT   264
EHI_035500       PANNIKVMLADFGNANWIEKRFTNDIQTRQYRCPEVMLGLHWGCPADVWSHACMIFELLT   261
ENU1_094620      PANNIKVMLADFGNANWIEKRFTNDIQTRQYRCPEVMLGLHWGCPADVWSHACMIFELLT   261
ScSky1_1Q8Y_A    PENLIQIKIADLGNACWYDEHYTNSIQTREYRSPEVLLGAPWGCGADIWSTACLIFELIT   230
HsSprk2_2X7G_A   NADKIRVKIADLGNACWVHKHFTEDIQTRQYRSIEVLIGAGYSTPADIWSTACMAFELAT   280
                   : *:: :**:*** * .:::*:.****:**. **::*  :.  **:** **: *** *
```

B

```
EHI_008150       -MILFYTVVTLCFAIQNNNDLRQILQRSDFVINSADKFIDDFRNTLKKSTIATEMFIQKE   59
EMO_117840       MFFLFVLFFYLCCCIENKKDLRQLLQRSDFIINSADKFIDDYRNTLSKSKAATDMFKTKE   60
ENU_104350       -MILFYTVVTLCFAIQNNNDLRQILQRSDFVINSADKFIDDFRNTLKKSNTATEMFIQKE   59
                  ::**  .. ** .*:*::****:******:**********:****.**. **:**  **

EHI_008150       PPKLHKKEQIKRIHSIQKSPTNRKYIKSTQPL-ITSHEPMKITSLNQRITEFPYFTPMLN   118
EMO_117840       VPKPHEKETIKRLSSPRTKPVVRKYIRPTRIQHSNPNQPLRISTINQRITEFPYFTHVLN   120
ENU_104350       PPKLHKKEQIKRINSIQKSPTNRKFIKSTQSL-ITSHEPMKITSLNQRITEFPYFTPILN   118
                  ** *:** ***: * :..*. **:*: *:     . ::*::*::*********** :**

EHI_008150       VNEFTQTKDKCNPHAPSYGYYIPIVIPPKKMLRVSTCNKDTTVFTPISVTYNEQCLQVIQ   178
EMO_117840       VSEFTQTEDKCNVGGPSYGYYIPIIIPPKKMLRVSTCNEDTTVFTPISVTYNGQCLQLIQ   180
ENU_104350       VNEFTQTKDKCNPHAPSYGYYIPIVIPPKKMLRVSTCNKDTTVFTPISVTYNEQCLQVIQ   178
                 *.*****:****   .*********:*************:************ ****:**

EHI_008150       RKCRLWNGNIIEYLPKGNKGMEAIVRVGASNVKNATVRITVYTVPITEKNKLKLEQGHVM   238
EMO_117840       RKCRRWSGNIIEYLPKSENGMEAIVRVGASNTKNAKVRITIYTVPITEKNKMKLQQGHVM   240
ENU_104350       RKCRLWNGNIIEYLPKGNKGMEAIVRVGASNVKNATVRITVYTVPITEKNKLKLEQGHVM   238
                 **** *.*********.::***********.***.****:**********:**:*****

EHI_008150       RGNVQQSTVKVTTKDGDIVPRIDFKNNTQTTHTS--------DNIISQPNHTIQLLEKDF   290
EMO_117840       RGNSKNTKVKVTTRKGDIVPTIEFNNSSKTSQTPTTDAQPFRDVSKTGPNHSIQLLVKNT   300
ENU_104350       RGNVQQSTVKVTTKDGDIVPRIDFKNNTKTTHTS--------DNIISQPNHTIQLLEKDF   290
                 *** :::.*****:****  *:*:*.::*::*         *   : ***:**** *:

                         TM
                      ___________
EHI_008150       HKMGGWFKVCIAILISILVLTVGVIIFGSFRESQLPEQYSPF   332
EMO_117840       QKMGGWFKVCIAIVISVVILTVGVIIFGSFRESRQPKQYAPF   342
ENU_104350       HKMGGWFKVCIAILISILVLTVGVIIFGSFRESQLPEQYSPF   332
                 :************:**::**************: *:**:**
```

Fig S9 Amino acid alignment of EHI_035500 (A) and EHI_008150 (B), and their homologs from other organisms. (A) Amino acid alignment of EHI_035500 and homologs from *E. invadens* (EIN_093630), *E. nuttalli* (ENU1_094620), yeast Sky1 (SCKG_2231), and human Sprk2 (2X7G_A). The residue that was found to be mutated in miltefosine-resistant *E. histolytica* clones is indicated with a red arrow. (B) Amino acid alignment of EHI_008150 and homologues from *E. moshkovskii* (EMO_117840) and *E. nuttalli* (ENU_104350). The residue that was found to be mutated in miltefosine-resistant *E. histolytica* clones is indicated with a red arrow. The C-terminal transmembrane region is marked with a black horizontal line (TM).

Fig S10, Saito-Nakano et al.

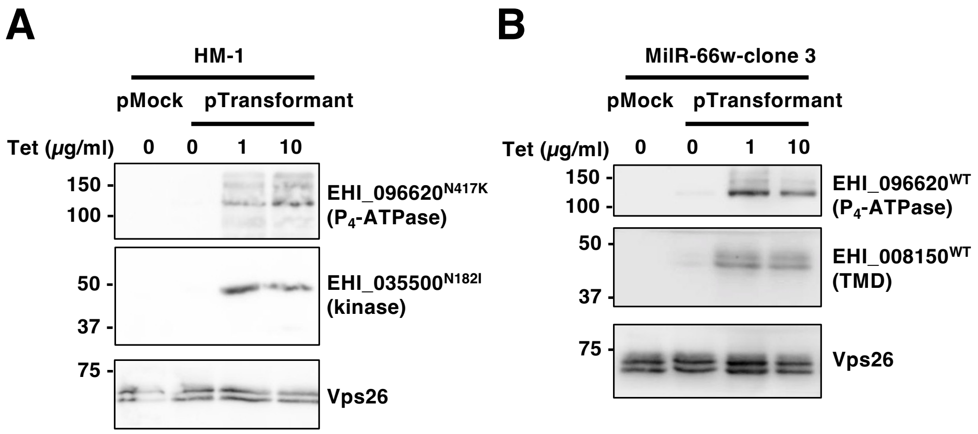

Fig S10 Immunoblot analysis of *E. histolytica* parental (A) and miltefosine-resistant (B) strains that express either mutated miltefosine resistance-associated gene products. (A) Transfomant strains that expressed EHI_096620^{N417K} or EHI_035500^{N182I} fused with the C-terminal HA-tag were created from wild-type HM-1. Tetracycline was added at indicated concentrations for 18h. Total cell lysates from EHI_096620^{N417K} or EHI_035500^{N182I}-expressing strain (pTransformant) and mock-transfected control (pMock) strain were subjected to SDS-PAGE and immunoblot analysis using anti-HA and anti-Vps26 antibodies as an internal control. The 120 and 43 kDa bands were detected corresponding to EHI_096620^{N417K} and EHI_035500^{N182I}, respectively. The transformant trophozoites expressing EHI_008150^{L20F} failed to be established despite repeated attempts. (B) Transfomant strains that expressed EHI_096620^{WT} or EHI_008150^{WT} fused with the C-terminal HA-tag were generated from miltefosine-resistant MilR-66w-clone 3. Tetracycline was added at indicated concentrations for 18h. Immunoblot analysis using total cell lysates from EHI_096620^{WT}- and EHI_008150^{WT}-expressing strains revealed the 120 and 38 kDa bands corresponding to EHI_096620^{WT} and EHI_008150^{WT}, respectively. The transformant trophozoite expressing EHI_035500^{WT} failed to be established despite the repeated lipofection.
